# On the complexity of Engh and Huber refinement restraints: the angle τ as example

**DOI:** 10.1107/S0907444910040928

**Published:** 2010-11-16

**Authors:** Wouter G. Touw, Gert Vriend

**Affiliations:** a Centre for Molecular and Biomolecular Informatics (CMBI), Nijmegen Centre for Molecular Life Sciences (NCMLS), Radboud University Nijmegen Medical Centre, 6525 GA 26 Nijmegen, The Netherlands

**Keywords:** protein structure, protein geometry, stereochemical parameters, restraints, N—C^α^—C bond angle

## Abstract

The Engh and Huber parameters for bond lengths and bond angles have been used uncontested in macromolecular structure refinement from 1991 until very recently, despite critical discussion of their ubiquitous validity by many authors. An extensive analysis of the backbone angle τ (N—C^α^—C) illustrates that the Engh and Huber parameters can indeed be improved and a recent study [Tronrud *et al.* (2010 ▶), *Acta Cryst.* D**66**, 834–842] confirms these ideas. However, the present study of τ shows that improving the Engh and Huber parameters will be considerably more complex than simply making the parameters a function of the backbone ϕ, ψ angles. Many other aspects, such as the cooperativity of hydrogen bonds, the bending of secondary-structure elements and a series of biophysical aspects of the 20 amino-acid types, will also need to be taken into account. Different sets of Engh and Huber parameters will be needed for conceptually different refinement programs.

## Abstract 2

The angle τ (backbone N—C^α^—C) is the most contested Engh and Huber refinement target parameter. It is shown that this parameter is ‘correct’ as a PDB-wide average, but can be improved by taking into account residue types, secondary structures and many other aspects of our knowledge of the biophysical relations between residue type and protein structure.

## 1. Introduction

Engh and Huber determined standard bond-length and bond-angle parameters (Engh & Huber, 1991 ▶, 2001 ▶) from crystal structures in the Cambridge Structural Database (CSD; Allen, 2002 ▶). They analyzed fragments equivalent to amino-acid side chains and the polypeptide backbone. The Engh and Huber (EH) values are applied as stereochemical restraint targets in most macromolecular refinement programs.

Two important assumptions have silently become accepted as facts by the use of the EH libraries in protein structure refinement. The first is that the stereochemistry in the peptide fragments in the CSD is the same as that in proteins and the second is that the stereochemical restraints are not a function of the environment.

Restraint targets for proteins are ideally derived from protein structures refined at atomic resolution without the use of any restraints. Only a very small number of such structures had been deposited in the Protein Data Bank (PDB; Bernstein *et al.*, 1977 ▶; Berman *et al.*, 2000 ▶) at the time when Engh and Huber first defined the restraint targets. Geometrical parameters could not, of course, be extracted from low- and intermediate-resolution structures as these were biased by the geometric restraints that were (necessarily) applied during the refinement. Therefore, at the time the peptide fragments in the CSD constituted the best source for defining target values for protein geometric restraints (EU 3-D Validation Network, 1998 ▶).

The EH parameters have been discussed ever since their introduction. Laskowski observed values other than those of Engh and Huber for bond lengths and angles in a data set consisting of the 186 ‘best’ structures in the PDB in 1993 (Laskowski *et al.*, 1993 ▶) and found that the refinement software used was a significant factor. Although they did not explicitly mention it, they observed that the N—C^α^ bond length depended on the residue type. Parameters calculated from unrestrained full-matrix refinement models of crambin (Stec *et al.*, 1995 ▶) and ColE1 repressor of primer (ROP; Vlassi *et al.*, 1998 ▶) showed statistically significant differences to the EH parameters. In both cases, the authors suggested adjustment of the EH parameters by taking into account parameters determined from atomic resolution protein structures. More recently, a correlation between the refinement program and the r.m.s. deviations from bond-length targets was observed (Jaskolski *et al.*, 2007*a* ▶). In the same study, an analysis of ten ultrahigh-resolution structures suggested that several EH main-chain target values should be adjusted and weighted differently. This suggestion initiated a dispute over the adjustment of stereochemical restraints and their weights in refinement (Jaskolski *et al.*, 2007*a* ▶,*b* ▶; Stec, 2007 ▶; Tickle, 2007 ▶; Karplus *et al.*, 2008 ▶).

The concept that the EH parameters should be independent of the stereochemical environment has also been questioned. The backbone torsion angles were found to correlate with the backbone geometry in empirical and theoretical studies (Karplus, 1996 ▶; Jiang *et al.*, 1997 ▶; Van Alsenoy *et al.*, 1998 ▶). A stereochemical analysis of the 0.87 Å resolution RNase A structure revealed significantly different N—C^α^—C (τ) angle values for α-helices and β-strands (Esposito *et al.*, 2000 ▶).

The number of atomic resolution structures has increased enormously since the introduction of the EH parameters, so that they can now be used to reinvestigate geometric protein parameters in a statistically meaningful way. The analyses of a large number of atomic resolution structures indeed con­firmed that ideal geometry is more complex than the context-independent geometry of the EH target values (Berkholz *et al.*, 2009 ▶). It has been suggested that refinement methods should incorporate ‘ideal geometry functions’ that define the ‘ideal’ target values as a function of ϕ, ψ (Berkholz *et al.*, 2009 ▶; Karplus, 1996 ▶; Karplus *et al.*, 2008 ▶). Tronrud *et al.* (2010 ▶) recently re-refined a series of ferredoxin reductase data sets using their so-called CDL (Berkholz *et al.*, 2009 ▶) library of (ϕ, ψ)-dependent standard values for the protein-backbone bond lengths and bond angles. The CDL target values for angles vary by as much as 3.5° from the EH values. They found that re-refinement did not improve the *R* factors, but did improve the overall geometry.

A geometrical parameter must take into account all factors that can influence it and great care should be taken to avoid new biases, especially when the parameter will be used in refinement methods. We studied the backbone angle τ (N—C^α^—C) in great detail because the normality score of this angle is one of the checks in the *WHAT_CHECK* software (Hooft, Vriend *et al.*, 1996 ▶; Hooft, Sander & Vriend, 1996 ▶) and when calling something ‘not normal’ we must know very well what is ‘normal’. We started by asking which parameters could influence τ. The residue type, ϕ, ψ angles and refinement software have already been mentioned. Looking at elementary biophysical aspects of amino acids, we came up with several other factors. The β-branched nature of Val, Ile and Thr, the possibility that several residue types (most prominently Ser, Asp and Asn) can form hydrogen bonds to their own local backbone, the cooperative nature of the hydrogen-bond pattern inside regular secondary structures and perhaps even the global bending of entire secondary-structure elements all seem to be good candidates to have an influence on τ.

Our analysis shows that all these factors are part of a large and complex set of factors that contribute to τ and that investigating their individual influences is not straightforward.

## 2. Methods

The PDBFINDER database (Hooft, Sander, Scharf *et al.*, 1996 ▶) release of 19 January 2010 was used to collect administrative information about PDB entries, such as the experimental method, resolution and refinement software used. The *WHAT IF* web services (Hekkelman *et al.*, 2010 ▶) were used to determine structure-wide parameters such as Ramachandran plot score (Hooft *et al.*, 1997 ▶) and packing quality (Vriend & Sander, 1993 ▶) and to determine parameters at the residue level such as τ, *DSSP* (Kabsch & Sander, 1983 ▶) secondary structure and area in the Ramachandran plot. A PostgreSQL (v.8.3.10) database was constructed to store the administrative and geometrical information. The database has separate sets of tables for PDB-file-wide data and for data at the level of the individual amino acid. In cases in which multiple refinement programs were mentioned in a PDB entry, we used common sense to guess which one was used last and thus left the strongest mark on the fine geometric detail. For example, we guessed that *REFMAC* (Murshudov *et al.*, 1997 ▶) was used in the final stage of refinement if both *X-PLOR* (Brünger, 1992 ▶) and *REFMAC* were mentioned. All observed combinations of refinement programs, and our decision on which one was used last, are described in Table S1 of the supplementary material^1^.

Molecular graphics were produced with *YASARA* (http://www.yasara.com/).

The *PISCES* data-set culling server (Wang & Dunbrack, 2003 ▶) was used to select sequence-unique structures.

We used *WHAT IF*’s internal database (Vriend, 1990 ▶) to calculate Ramachandran plots for residues at the beginning of an α-helix and for residues in the middle of an α-helix.

The statistical language R (R Development Core Team, 2008 ▶) was used to perform statistical tests and to create dot plots. All statistical tests in this study were two-sided two-sample t-tests performed using the R function ‘t.test’. The dot plots were created using the R package ‘lattice’ (Sarkar, 2007 ▶).

## 3. Results

This study was based on a data set comprising >50 000 PDB files. These files were selected using the criteria listed in Table 1 ▶. More than 23 million residues were selected for further study. The criteria for using a residue are listed in Table 2 ▶.

Structures were divided into five resolution bins: <1.0, 1.0–1.5, 1.5–2.0, 2.0–2.5 and ≥2.5 Å. Selected residues were grouped by residue type and by the three secondary-structure classes H (α-helix), S (β-strand) and C (everything else, which we will refer to from here on as ‘loop’). This resulted in initially 5 × 20 × 3 = 300 groups for which τ was analyzed. By the term ‘all residues’ we mean 18 of the 20 canonical amino-acid types, excluding Gly and Pro. Similarly, average values are always taken over these 18 residue types, unless mentioned otherwise.

Because of the enormous number of counts in each category, almost all differences are statistically significant, with *p*-­values much better than 0.01. For example, the τ angles for residues in a β-strand at 1.5–2.0 and 2.0–2.5 Å resolution are 109.2 ± 3.0° and 109.5 ± 3.1°, respectively. These numbers are obtained from 1.2 million and 1.7 million observations, respectively, so that the significance of this small angular difference is very high (p << 10^−10^). Even in the highest resolution bin, which contains the fewest observations, many differences are still highly significant. For example, the difference between Glu, H (111.5°, 817 counts) and Glu, C (111.1°, 504 counts) is significant, with *p* = 0.001. On the other hand, the difference between Lys, H and Lys, C is 0.1° in the highest resolution class and owing to the low number of counts in this bin this difference is not significant (*p* = 0.381, 674 counts). According to Student’s t-test, a 0.1° difference in the mean of two Gaussians both with σ = 2.5° is significant with *p* = 0.01 if the number of observations is 8300. For our data set, this means that the number of observations in all bins apart from that with the highest resolution is large enough to make differences of 0.1° in the average τ angle significant. All differences that we will mention are significant at *p* = 0.01 or better, unless specified otherwise.

**Figure 1:**
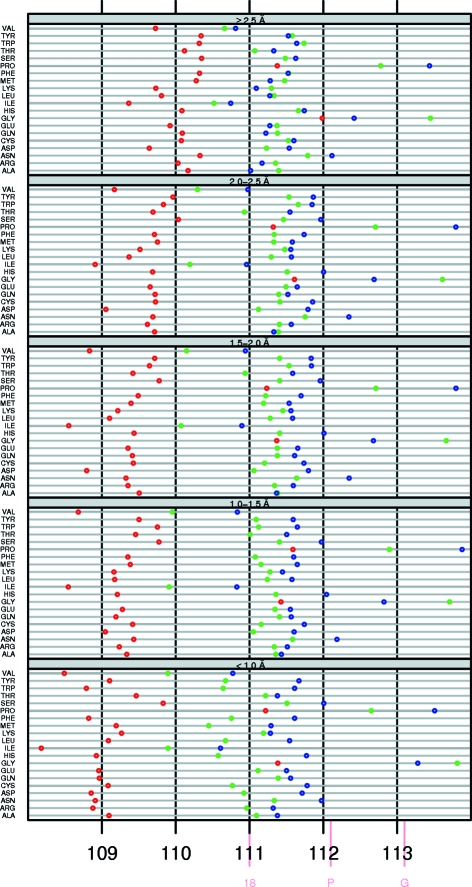
The average τ angle per residue type as a function of resolution (from top to bottom, ≥2.5, 2.0–2.5, 1.5–2.0, 1.0–1.5 and <1.0 Å) and secondary structure (red, β-sheet; green, loop; blue, α-helix). The pink tick marks on the horizontal axis indicate the EH values (Engh & Huber, 2001 ▶) for Gly (G), Pro (P) and the 18 other amino-acid types (18).

Fig. 1 ▶ shows the τ angles as function of secondary structure, residue type and resolution bin. Just like Karplus (1996 ▶) and Esposito *et al.* (2000 ▶), we observed that τ in a β-strand is significantly lower than τ in an α-helix or loop. We see that this is true for all residue types in all resolution bins. τ is generally slightly higher in an α-helix than in a loop.

The τ value in β-strands strongly depends on the resolution. In the highest resolution bin the average τ is 109.0 ± 1.9°, while it is 110.0 ± 3.3° if the resolution is worse than 2.5 Å. In α-­helices τ tends to be slightly lower at the lowest resolution (111.3 ± 2.5°) than at the highest resolution (111.4 ± 1.4°), while τ in loops is a little lower in the highest resolution bin (111.0 ± 2.4°) than in the other resolution classes (111.3 ± 3.4°). The most recent EH value is 111.0° for all residues (Table 3 ▶). As expected, τ converges to this value at low resolution, especially in β-strands. At low resolution, the low amount of X-ray data causes the target restraints to be applied with more emphasis during refinement than at high resolution.

Several groups have performed analyses on culled data sets in order to avoid biases in their studies (*e.g.* Laskowski *et al.*, 1993 ▶; Holmes & Tsai, 2004 ▶; Jaskolski *et al.*, 2007*a* ▶; Berkholz *et al.*, 2009 ▶). We performed several data-selection experiments in which we measured τ either after the removal of poor structures or after sequence-identity culling.

The same trends were observed when we removed the worst 25% of the structures (36% of the residues) according to a series of *WHAT_CHECK* validation scores. Structures were discarded if more than 5000 amino acids were present in the structure, if more than 25% of the residues had missing atoms, if more than 10% of the amino acids had missing backbone atoms, if the resolution was worse than 3.5 Å, if the Ramachandran *Z* score was below−5.0, if the χ_1_/χ_2_ correlation *Z* score was less than −5.0, if the root-mean-square *Z* scores (r.m.s.*Z*) for bond lengths or bond angles were smaller than 0.25 or larger than 1.50, if multi-model refinement had been applied to the structure or if the packing quality was worse than commonly observed for homology models. We did not observe significant differences between the results obtained from the full data set and the reduced data set. This shows that the observed τ values are not dictated by a series of poor (or old) structures but are the genuine result of the refinement process.

The characteristics of τ also were not altered when we culled the data set using sequence identity. Selection of only proteins that are sequence-unique at the 90% or the 25% sequence-identity level negatively affected the counting statistics, but had no significant influence on the observed averages for τ. This was to be expected because almost all structures in the PDB are refined by a different person who might have used a different program and might have used different settings. Thus, the heterogeneity in unculled data sets is also observed in culled data sets.

Gly and Pro systematically have a higher τ than other amino acids in all secondary-structure types at all resolutions. Gly in loops has a higher τ than Pro in loops, whereas in α-­helices this is the other way around. The aberrant geometry of Gly and Pro was previously noted by Engh & Huber (1991 ▶).

**Figure 2:**
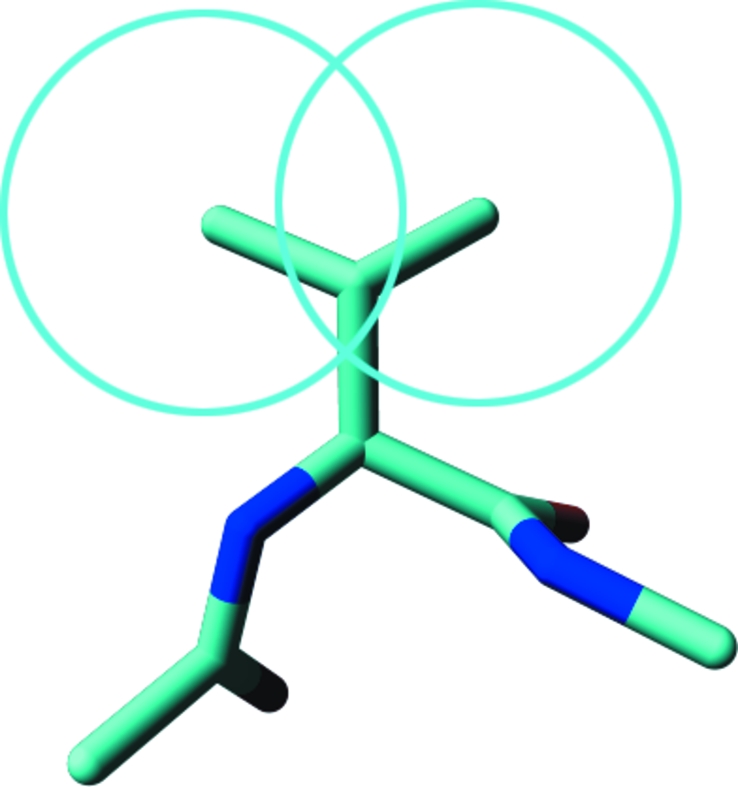
Both C^γ^ atoms in Val push against their own backbone. The two circles that are centred on the C^γ^ atoms have a radius of about 1.8 Å, reflecting a commonly used van der Waals radius for these CH_3_ groups.

The C^γ^ atoms of both Ile and Val (Fig. 2 ▶) ‘push’ the backbone, which must result in a smaller τ. Indeed, these residues typically have a much lower τ than other residues. In the third β-­branched residue Thr, τ is closer to the τ of ‘normal’ residue types. Berkholz *et al.* (2009 ▶) concluded that ‘Thr behaves more like a general residue because of stabilizing side chain–backbone hydrogen bonds’. We also observe that the reduced τ value seen for the β-­branched Val and Ile is not seen for Thr. We explicitly looked for hydrogen bonds for the Thr O^γ^ atoms, but could find no trends, perhaps because Thr does not easily form hydrogen bonds with its own local backbone.

To find out whether the observed τ is influenced by the refinement software, we analyzed τ separately for structures refined with *CNS* (Brünger *et al.*, 1998 ▶; 18 000 PDB files), *REFMAC* (21 000 PDB files), *X-PLOR* (7000 PDB files) and *SHELXL* (Sheldrick, 2008 ▶; 2000 PDB files). Other refinement programs have not been used often enough to allow any meaningful statistics. More than 80% of the whole data set (76% of the entries) had been refined with *CNS* or *REFMAC*.

Some of the early refinement programs have been replaced by newer and better ones. We left out most of the old programs in our analysis. *X-PLOR* has been superseded by *CNS* and is no longer used very frequently; it was used to refine only 0.25% of all X-ray structures in 2009. It was nevertheless included because 13.6% of all PDB files in our data set had been refined with *X-PLOR*.

**Figure 3:**
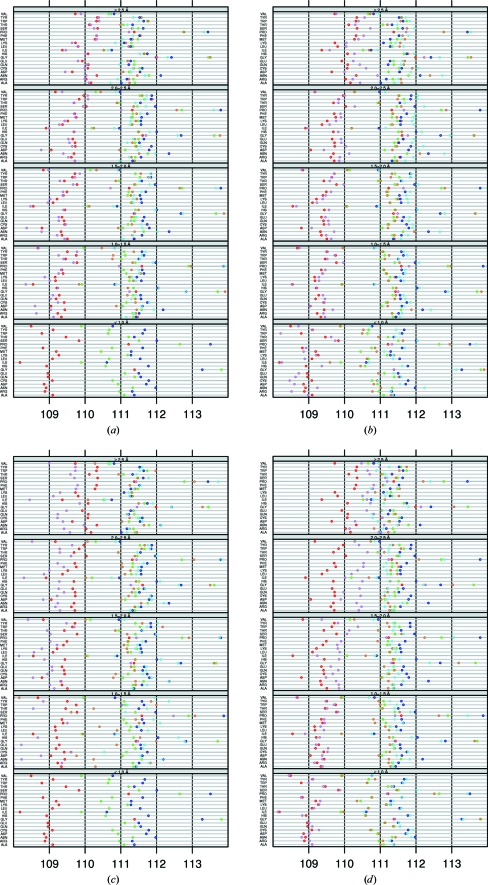
τ as a function of refinement program. (*a*) *CNS*, (*b*) *REFMAC*, (*c*) *X-PLOR*, (*d*) *SHELXL*. The subdivisions in resolution and secondary structure are the same as in Fig. 1 ▶. The red and blue circles are the same in all four panels and are the same as in Fig. 1 ▶. The τ angles that resulted from structures refined with the indicated refinement software are shown in pink (sheet), brown (loop) and light blue (helix). We gave all plots the same dynamic range on the *x* axis for clarity. Points on the vertical axes actually fall outside the range of the *x* axis. The true values are available from the associated web pages at http://swift.cmbi.ru.nl/gv/whatcheck/HTML/TAU/.

In structures refined with *CNS* (Fig. 3 ▶ *a*) τ is generally lower than in structures refined with *REFMAC* (Fig. 3 ▶ *b*). *X-PLOR* (Fig. 3 ▶ *c*) produces even lower τ angles, especially in β-strands. Compared with other programs, *SHELXL* (Fig. 3 ▶ *d*) shows more convergence to the EH value of 111.0° towards lower resolution.

*REFMAC* and *CNS* show a consistent increase or decrease of τ as a function of resolution for many residue types. In some cases a different pattern is observed for specific residue types. *CNS* gives a relatively low τ value for Asp in strands, while τ for Asn in helices is relatively high in structures refined with *REFMAC*. Understanding where these small anomalies come from seems hardly possible at present.

**Figure 4:**
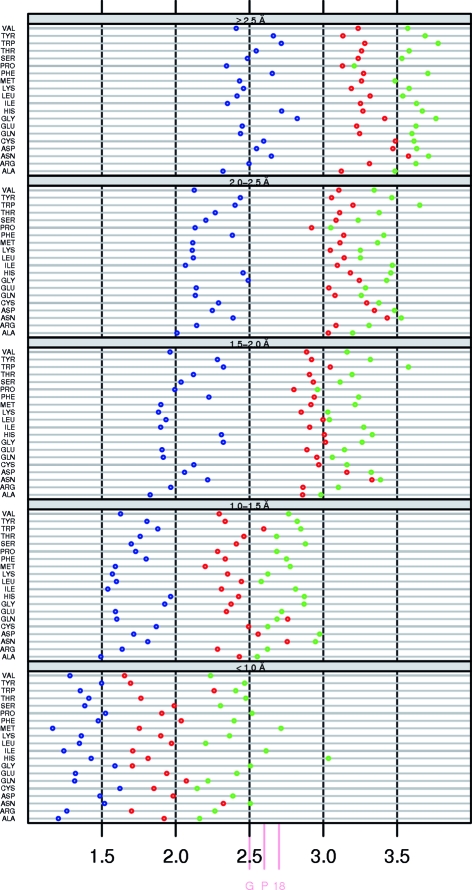
The standard deviation on τ per residue type as a function of secondary structure and resolution. Secondary-structure colours and resolution bins are the same as in Fig. 1 ▶. The pink tick marks on the horizontal axis indicate the EH values (Engh & Huber, 2001 ▶) for Gly (G), Pro (P) and the 18 other amino-acid types (18).

**Figure 5:**
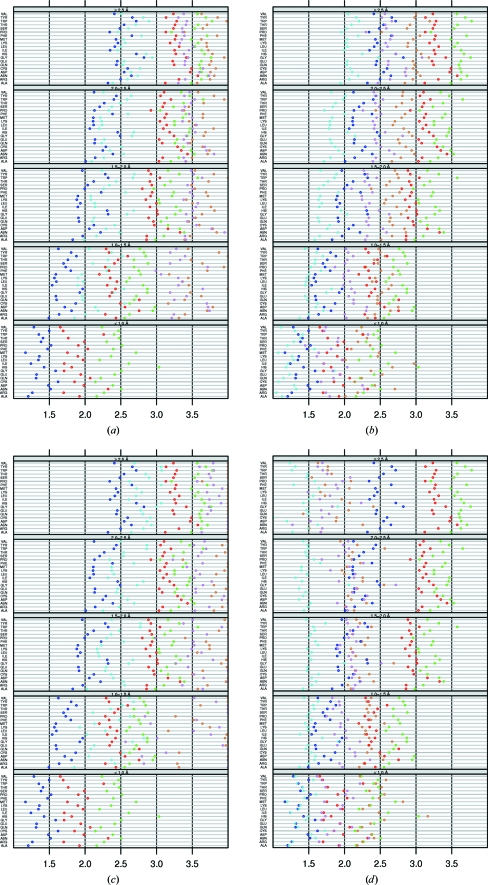
The standard deviation on τ of the four refinement programs (*a*) *CNS*, (*b*) *REFMAC*, (*c*) *X-PLOR* and (*d*) *SHELXL*. Resolution bins and secondary-structure colouring are as in Fig. 4 ▶. The global σ, indicated in red and blue, is the same in all four panels and is the same as in Fig. 4 ▶. The σ values that resulted from structures refined with the indicated refinement software are shown in pink (sheet), brown (loop) and light blue (helix). We gave all plots the same dynamic range on the *x* axis for clarity. Points on the vertical axes actually fall outside the range of the *x* axis. The true values are available from the associated web pages.

The EH parameters not only provide refinement target values for τ but also their standard deviations. These standard deviations are actually equally as important as the mean values because they determine the relative strengths of the restraints in refinement and the allowed deviations in structure validation. The observed standard deviations are lowest in helices and highest in loops (Fig. 4 ▶). The average standard deviations increase towards lower resolution. This trend is observed for all secondary structures and all residue types. This trend seems to be counterintuitive, as a lower standard deviation would be expected when the contribution of the target restraints becomes more important. However, the solution space of X-ray structure determination contains many local minima. A major cause of the existence of these local minima is the use of torsion-angle restraints on the side chains; these restraints are available in *REFMAC* and *CNS* but not in *SHELXL*. The local minima caused by the target restraints are probably more prominent at low resolution than at high resolution, so that in many low-resolution cases the local structure will remain in a (wrong) local minimum. These ideas are supported by the observation that structures refined with *SHELXL* do converge more towards the EH values (Fig. 3 ▶ *d*) and have a decreasing standard deviation towards lower resolution (Fig. 5 ▶ *d*). Indeed, none of the other programs showed this trend.

The standard deviation is a function of resolution for *SHELXL* and *REFMAC* (Fig. 5 ▶ *b*), although reverse trends are observed for these programs. In structures refined with *CNS* (Fig. 5 ▶ *a*) and *X-PLOR* (Fig. 5 ▶ *c*) the standard deviation on τ is generally higher than the average and is much less a function of the resolution. A similar impact of the refinement program has been observed previously (Laskowski *et al.*, 1993 ▶; Jaskolski *et al.*, 2007*a* ▶), although in these studies a slightly different set of refinement programs was analyzed and a rather different analysis approach was used. In our study, it is shown that in addition to resolution, secondary structure and residue type, refinement program is also a factor which influences τ.

**Figure 6:**
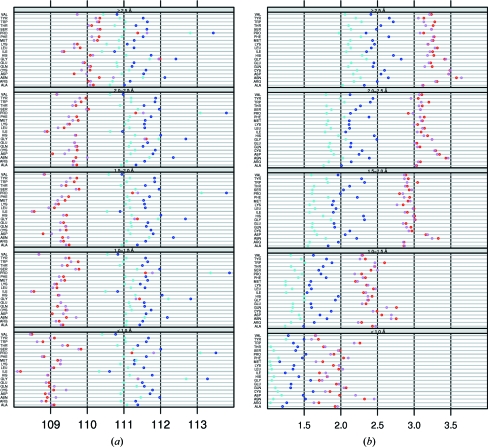
The average τ (*a*) and standard deviation on τ (*b*) for residues in the middle of a secondary-structure element compared with the average values for the whole element. Whole-helix and whole-strand values are shown in blue and red, respectively, and are the same as in Figs. 1 ▶ and 4 ▶. Values for the middle of the secondary-structure elements are shown in light blue (helix) and pink (strand). For clarity, the same *x*-axis ranges are used as in Figs. 1 ▶ and 4 ▶. Some residues in the middle of a helix have a standard deviation lower than 1.0° in the highest resolution bin. The light blue points on the *x* axis indicate these values (top to bottom): Val, 0.91; Trp, 0.95; Pro, 0.95; Ala, 0.94.

Residues at the beginning of a secondary-structure element experience different forces than residues in the middle of a secondary-structure element. For example, the backbone of a residue at the first few positions of an α-helix only donates a hydrogen bond, while at a position further in the helix the backbone both donates and accepts a hydrogen bond. Additionally, small deviations from the ideal backbone angles will cause much less structure disturbance near the ends of secondary-structure elements than in the middle. To investigate whether the cooperative effect of the hydrogen-bonding pattern inside secondary-structure elements influences τ, we compared the overall τ values in α-helices and β-strands with residues in the middle of an α-helix and in the middle of a β-­strand. The middle of an α-helix is defined as being at least five residues away from either end of the helix and the middle of a β-strand is defined as being at least two residues away from either end of the strand. Averages and standard deviations for helices and strands are shown in Figs. 6 ▶(*a*) and 6 ▶(*b*), respectively. For both helices and strands τ is lower in the middle than at the ends. Inside an α-helix τ is more than 0.5° lower than the average τ of all α-helical residues. Inside β-­strands τ is about 0.1° lower than the average τ of all residues in a β-strand. As the whole data set includes ends and middle sections of secondary structures, the actual differences between the middle sections of secondary structures and their ends is actually even larger than these values. The standard deviations also are smaller in the middle of secondary-structure elements than at their ends. Both effects are stronger in α-­helices than in β-strands.

Most residues have backbone ϕ, ψ angles that fall in the areas of the Ramachandran plot commonly called ‘the helix area’ (ϕ, ψ ≃ −60, −40°) or ‘the strand area’ (around ϕ, ψ ≃ −150, 150°). This is also true for residues that are in a loop or turn according to *DSSP*. An α-­helix, for example, is only observed if a series of residues in a row have ϕ, ψ ≃ −60, −40° and if the hydrogen bonds are all of the proper type for an α-helix (O_*i*_→N_*i*+4_—H), while several types of β-turn consist of a residue with helical ϕ, ψ followed by a residue with strand-like ϕ, ψ. As it seems likely that a residue with helical ϕ, ψ angles in a loop feels different forces from a residue in the middle of a helix, we decided to compare the τ angles of these two classes. The cooperative effect for residues in the middle of regular secondary structures will not be felt by nonhelical residues in the helix area or nonstrand residues in the strand area. The absence of co­operative forces in loops might also explain the observation that the difference in τ between Val/Ile and the other 16 non-Gly, non-Pro residue types is larger in loops than in helices and strands (Fig. 1 ▶). We defined the β-strand area (B) by ϕ < −4° and ψ > 100°. The α-helix area (A) was defined as −120 < ϕ < −40° and −60 < ψ < 20°. The left-handed helix area (L) was defined as 40 < ϕ < 120° and −20 < ψ < 60°. The rest of the Ramachandran plot is called U. These areas are a little wider than the Ramachandran plot suggests, especially in the corners. This does not cause problems because the corners of these areas are barely populated anyway. Each residue now has a double code: one for its secondary structure (H, S, C) and one for its area in the Ramachandran plot (A, B, L, U).

**Figure 7:**
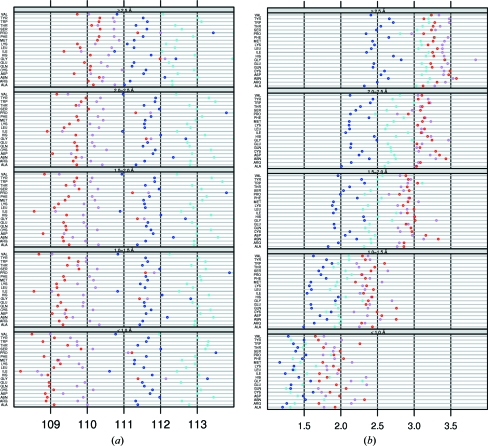
The average τ (*a*) and standard deviation on τ (*b*) for residues in a helix (‘H’, dark blue), in a strand (‘S’, red), in a loop with ϕ, ψ angles in the helix area (‘C_A’, light blue) and in a loop with ϕ, ψ angles in the strand area (‘C_B’, pink). Dark blue and red circles are the same as in Figs. 1 ▶ and 4 ▶. We chose to use the same *x*-axis range as in the other figures for clarity. Gly and Pro τ angles that are higher than 114° are shown at 114°. The true values are available from the associated web pages.

Fig. 7 ▶ shows a comparison of τ angles in α-helices and β-­strands with residues that are in a loop according to *DSSP* but with ϕ, ψ angles in the helix area and the strand area, respectively.

Residues in a loop (according to *DSSP*) with local ϕ, ψ angles in the helix area of the Ramachandran plot (‘C_A’) have a more than 1.0° higher τ than residues in an α-helix (Fig. 7 ▶ *a*). τ in the helix area of α-helices (‘H_A’) is not different from the superset ‘H’ (and only about one in 1000 of these residues fall in the left-handed helix area or the strand area, as can be seen from the material available from the associated web pages at http://swift.cmbi.ru.nl/gv/whatcheck/HTML/TAU/). ‘C_A’ has an increasingly higher standard deviation than ‘H’ towards lower resolution. Loops in the β-­strand area (‘C_B’) have a significantly higher τ of about 0.5° than β-strands (‘S’), while ‘S_B’ is not different from ‘S’ (and less than one in ten residues in β-­strands fall outside the β-strand area). Our results indicate that τ is more a function of secondary structure than of backbone torsion angles.

**Figure 8:**
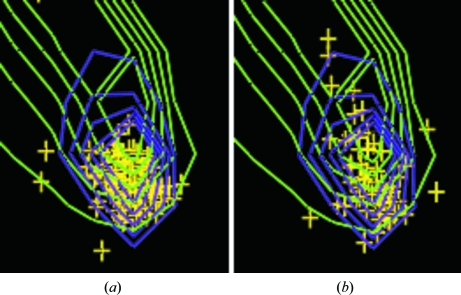
Ramachandran plots of the helical area for (*a*) residues five positions away from both ends of helices that are nine residues long or longer and (*b*) residues at one of the first three positions of an α-helix. The contour lines are for the 18 normal residues in a helix (purple) and in a loop (green), contouring at 50–90% of what is observed in the *WHAT IF* database of 500 sequence-unique high-quality X-ray structures solved at 1.4 Å resolution or better (Hooft *et al.*, 1997 ▶).

The combination of secondary structure and the region in the Ramachandran plot influence τ. A two-dimensional Ramachandran plot cannot distinguish between, for example, residues in α-helices with helical ϕ, ψ angles and residues with helical ϕ, ψ angles not in α-helices, which is a pity as these groups have a different τ angle, as shown above. Neither can the difference be seen between the ends of helices and the middle. To illustrate this, we compared the Ramachandran plots of residues in the middle and in the N-terminal turn of an α-helix to see whether (at least) the difference in τ between these groups is reflected in the ϕ, ψ angle distribution. Fig. 8 ▶ shows the relevant part of Ramachandran plots for residues positioned inside an α-helix (position 5) and residues in the first turn of an α-helix (positions 1–3). The ϕ, ψ angle distributions show much overlap, but it is clear that the residues at position 5 in a helix cluster much more tightly around the core of the helical area.

## 4. Discussion

The angle τ in proteins depends on many factors and the single-value paradigm is too simple, as has been pointed out previously (see, for example, Karplus, 2008 ▶; Berkholz *et al.*, 2009 ▶; Tronrud *et al.*, 2010 ▶). Resolution, secondary structure, residue type and refinement program all influence τ significantly. Our results also indicate that things are actually much more complicated. Many bio­physical factors (residue type, secondary structure, location in the secondary structure, accessibility *etc*.) and computational factors [refinement software, target restraints (Tronrud *et al.*, 2010 ▶), refinement strategy and data resolution] influence this angle, although this does not always happen in the expected direction. The latter, of course, tells us more about our understanding of the biophysics of protein structures than about either the τ angles themselves or the way that they are refined in crystallography. We observe, for example, that the average τ angle for residues at the buried side of an α-­helix is on average 0.2° smaller than for residues at the solvent-accessible side. We also observe that in the middle of helices τ tends to be lower than at the ends, which may be caused by a combination of the cooperativity of the hydrogen bonds and the planarity of the peptide bonds. Sometimes the exact reasons for an observation are hard to understand, while the consequences for future activities are clear.

Many more parameters can be thought of that influence τ. We looked at the intramolecular contacts made by the residues, including in some cases the hydrogen bonds, but did not find statistically significant effects on τ. Tryptophan is very large and hydrophobic and thus normally makes many contacts that push and pull it into its conformation. The τ angle of tryptophan does not differ significantly from those of other residues, but it has a relatively high standard deviation. Asn and Ser, and to a lesser extent Asp, are known to form hydrogen bonds to the local backbone. Obviously, a hydrogen bond to the local backbone exerts a force on that backbone that will influence the τ angle. Asp often has a much lower τ than most other residues in β-strands, and Ser and particularly Asn have a high τ in helices. Structures refined with *REFMAC* show a rather high τ angle for Asn in all secondary structures and this thus also increases the τ angles observed for Asn in general. Inspection of the *REFMAC* dictionary revealed that the value given for Asn is 112.2 ± 2.8°. This differs significantly from the EH value of 111.2 ± 2.8° and suggests a typographical error in the *REFMAC* dictionary.

The leptokurtic τ distributions for residues in helices and loops, especially at low resolution, indicate that τ values are pulled towards the EH values during refinement. The τ angles in β-strands become closer to the EH value in lower resolution structures. However, these τ distributions are less leptokurtic than the corresponding ones in helices and loops, which is probably the result of two partly overlapping distributions stemming from the different hydrogen-bond patterns in parallel and antiparallel sheets.

We will need many more structures solved at better than 1.0 Å resolution that are refined without or with minimal use of refinement target constraints to find significantly distinct subgroups related to hydrogen-bonding differences, systematic van der Waals contacts or β-sheet arrangements (parallel *versus* antiparallel or edge strand *versus* central strand). The effects of β-sheet curvature on the τ angles still need to be studied.

Even with the enormous volume of data available today in the PDB, many effects cannot yet be determined with sufficient significance. Residues will have different EH parameters when they sit adjacent to a glycine, a proline or a *cis*-peptide to when they sit next to one of the other 18 residues in the *trans* conformation.

The use of (new) EH parameters should, of course, be different for different refinement programs. Programs that combine X-ray data with energy calculations (*e.g. X-PLOR* and *CNS*) should not, in principle, use a large number of different τ angles because all structure-dependent effects will already be introduced by the energy terms. Programs such as *REFMAC* and *SHELXL* (at medium and low resolution), for example, should use more differentiated τ angles. Care should be taken to not introduce any effects twice. The lower τ angles observed for valine and isoleucine are mainly caused by 1–4 repulsive interactions between the backbone and the side-chain γ atoms. Careful calibration will be required if such repulsive interactions are already an integral part of the software. Programs such as *CNS* and *X-PLOR* combine the X-­ray data terms with molecular-dynamics force-field terms in the simulated-annealing stage of the refinement process. It is noteworthy that they lead to τ angles that are closer to the EH values at low resolution than at high resolution. *CNS* and *X-­PLOR* use reduced van der Waals (or repulsive) radii for 1–­4 interactions. In light of this, it is gratifying to see that the actually observed average τ angles for the β-branched residues are rather independent of the refinement programs used. It might be a prudent step to lower the restraints on the τ angles (*i.e.* use a large standard deviation) in the refinement software at present in order to bridge the time until we know how to include differentiated τ-angle restraints in refinement software. Generally, it might be a suggestion to completely re-­evaluate the force fields used in molecular-dynamics-based refinement software, as these have improved significantly over the last decade.

Hydrogen bonds are shorter on the concave side of bent helices than on the convex side. In parallel, τ angles on the buried side of helices are smaller than τ angles on the accessible side. We do not know what is caused by what. Are the shorter hydrogen bonds pulling harder at the backbone or is the reduced space on the concave side easiest compensated by τ-angle shrinking? These questions are not so relevant for structure validation, but are very important for refinement software that uses molecular-dynamics energy terms.

The first experiments with differentiated EH parameters by Tronrud *et al.* (2010 ▶) suggest that many experiments along these lines will follow. The so-called CDL restraints used by Tronrud are a large step in the right direction, but they will be improved many times in the years to come, probably first by making the parameters secondary structure-dependent and (ϕ, ψ)-dependent rather than just (ϕ, ψ)-dependent. As long as all structural biologists keep faithfully depositing their experimental data, projects such as *PDB_REDO* (Joosten *et al.*, 2009 ▶) will re-refine the structures when, in due time, it becomes clear which is the ideal new EH target set to use.

## Supplementary Material

Supplementary material file. DOI: 10.1107/S0907444910040928/kw5028sup1.pdf

## Figures and Tables

**Table 1:** Selection criteria for PDB files

Selection parameter	Criterion
Experimental method	X-ray
PDB-file content	>25 amino acids
Refinement software	Must be mentioned
*DSSP* file	Must be determinable

**Table 2:** Selection criteria for residues

Selection parameter	Criterion
Type	Only 20 canonical amino-acid types
*DSSP* secondary structure	H, S or C/T/*etc*.
Position in structure	Not a C- or N-terminus; not next to a terminus; not next to Gly; not next to Pro
Backbone atoms	All four must be present
∠(N—C^α^—C) (τ) value	Within 10σ of group average

**Table 3:** The average τ with standard deviation (°) for Gly, Pro and the rest of the residue types (secondary-structure and resolution classes pooled)

	EH (1999)	EH2 (2001)	This study
Gly	112.5 ± 2.9	113.1 ± 2.5	113.1 ± 3.4
Pro	111.8 ± 2.5	112.1 ± 2.6	112.8 ± 3.0
Rest	111.2 ± 2.8	111.0 ± 2.7	111.0 ± 3.0

## Footnotes

1 Supplementary material has been deposited in the IUCr electronic archive (Reference: KW5028). Services for accessing this material are described at the back of the journal.
