# Supplementary material for: On the complexity of Engh and Huber refinement restraints: the angle τ as example
Source: Acta Crystallogr D Biol Crystallogr. 2010 Nov 16;66(Pt 12):1341–50. doi: 10.1107/S0907444910040928 (PMC2995724; doi:10.1107/S0907444910040928)
Supplement: Supplementary file 1 [file d-66-01341-sup1.pdf]

Supplementary material

Table S1 List of refinement program combinations that were found in PDB entries and our decision on which one most likely was used last.

PDB File	Interpretation	PDB File	Interpretation
**CNS + ARP**	CNS	**REFMAC + X-PLOR**	REFMAC
**CNS + NUCLSQ**	CNS	**RESTRAIN + ATOM**	RESTRAIN
**CNS + REFMAC**	REFMAC	**SHELXL + CNS**	SHELXL
**CNS + X-PLOR**	CNS	**SHELXL + NUCLSQ**	SHELXL
**EREF + PROLSQ**	PROLSQ	**SHELXL + X-PLOR**	SHELXL
**NUCLIN + NUCLSQ**	NUCLSQ	**TNT + NUCLSQ**	TNT
**PROFFT + NUCLSQ**	NUCLSQ	**TNT + PROLSQ**	TNT
**PROLSQ +AMORE**	PROLSQ	**TNT + X-PLOR**	TNT
**PROLSQ + ATOM**	PROLSQ	**X-PLOR + AMORE**	X-PLOR
**PROLSQ + EREF**	PROLSQ	**X-PLOR + ARP**	X-PLOR
**PROLSQ + X-PLOR**	X-PLOR	**X-PLOR + FRODO**	X-PLOR
**PROTIN + NUCLSQ**	NUCLSQ	**X-PLOR + NCS**	CNS
**REFMAC + AMORE**	REFMAC	**X-PLOR + NUCLSQ**	X-PLOR
**REFMAC + ARP**	REFMAC	**X-PLOR + QUANTA**	X-PLOR
**REFMAC + NCS**	REFMAC	**X-PLOR + RESTRAIN**	X-PLOR
**REFMAC + NUCLSQ**	REFMAC		

Other supplementary material

Additional material can be found at http://swift.cmbi.ru.nl/gv/whatcheck/HTML/TAU/.
